# The side effects and complications of percutaneous iodine-125 seeds implantation under CT-guide for patients with advanced pancreatic cancer

**DOI:** 10.1097/MD.0000000000009535

**Published:** 2017-12-29

**Authors:** Wei-Fu Lv, Dong Lu, Jing-Kun Xiao, Gauri Mukhiya, Zhong-Xiao Tan, De-Lei Cheng, Chun-Ze Zhou, Xing-Min Zhang, Zheng-Feng Zhang, Chang-Long Hou

**Affiliations:** a Department of Interventional Radiology, Anhui Provincial Hospital, The First Affiliated Hospital of University of Science and Technology of China, Hefei, Anhui, China; b Department of Radiology, BIR Hospital, National Academy of Medical Sciences, Kathmandu, Nepal.

**Keywords:** brachytherapy, complication, iodine-125 seed, pancreatic cancer, percutaneous implantation, side effect

## Abstract

**Purpose:**

The present study investigates the side effects and complications of computed tomography (CT)-guided percutaneous iodine-125 (I-125) seeds implantation for advanced pancreatic cancer.

**Methods:**

The clinical data were retrospectively analyzed for patients treated with implantation of I-125 seeds under CT-guide in our hospital from May 2010 to April 2015. The side effects and complications were collected and their possible reasons were analyzed.

**Results:**

A total of 78 patients were enrolled. The side effects were categorized as fever in 29 cases (37.18%), abdominal pain in 26 cases (33.33%), nausea and vomiting in 9 cases (11.54%), diarrhea in 5 cases (6.41%), and constipation in 4 cases (5.13%). Complications were composed of pancreatitis in 9 cases (11.54%), infection in 5 cases (6.41%), seed migration in 2 cases (2.56%), intestinal perforation in 1 case (1.28%), and intestinal obstruction in 1 case. The incidence of complication was 23.08% (18/78). The difference in incidence of complication was statistically significant between patients implanted with ≤27 seeds and those with >27 seeds (*P* = .032).

**Conclusion:**

The side effects and complications frequently occur in implantation of I-125 seeds for patients with advanced pancreatic cancer. More concern should be given to the patients treated by this technique.

## 1 Introduction

Pancreatic cancer is the 4th common cause of death among malignant tumors and is responsible for >250,000 deaths/year worldwide.^[1,2]^ Most patients with pancreatic cancer are diagnosed at the middle or advanced stages; only 10% to 20% of these patients are given the opportunity for radical surgery when they are diagnosed.^[3,4]^ Gemcitabine-based chemotherapy is an option that can improve survival rate and quality of life in patients with unresectable tumor. However, chemotherapy refractory and poor response are the major therapeutic challenges for these patients.^[5]^ Thus, minimally invasive therapies have come under development. The interstitial brachytherapy with Iodine-125 (I-125) seeds has been applied to treat pancreatic cancer at middle or advanced stages for >20 years.^[6–8]^ The efficacy of this therapy has been recognized and reported by several scholars,^[9,10]^ but few reports dealt with the adverse events following implantation. In the present study, the side effects and complications of I-125 seed implantation were analyzed to improve the safety of this therapeutic modality.

## 2 Materials and methods

### 2.1 Patient information and selection

This study was approved by the Ethics Committee of Anhui Provincial Hospital in China and abided with the Helsinki Declaration. A retrospective analysis of clinical data were performed in patients with pancreatic cancer at the advanced stages; these patients underwent implantation of I-125 seeds in Anhui Provincial Hospital from May 2010 to April 2015, the following inclusion criteria were used: patients with pancreatic cancer identified by pathological biopsy or 2 kinds of image examination combined with laboratory and clinical examination leading to a diagnosis of pancreatic cancer; patients at the IIB, III, or IV stage based on the Union for International Cancer Control 2002 standards; patients with medical records containing complete data of admission tests, as well as the course of treatment; percutaneous transhepatic catheter drainage or biliary stent implantation should be performed to relieve obstruction of the biliary tract and reduce the total bilirubin to essentially normal levels in patients with presence of jaundice before implantation of I-125 seeds; patients with preoperative enhanced computed tomography (CT) scans and complete laboratory tests; and patients who signed an informed consent form before the implantation.

The exclusion criteria were as follows: patients with serious cardiopulmonary function disorder, advanced dyspraxia; tumor sizes of ≥6 cm, as well as those with incomplete examination; and patients with mental disorders and those with other diseases.

### 2.2 Treatment plan protocol

Enhanced CT test was performed within 1 to 2 weeks before implantation of I-125 seeds to identify the cancer scope and boundary. Before I-125 seeds implantation under CT guide, the results of a recent CT scan were input into the treatment plan system (Beijing ASTRO Technology, Ltd., Beijing, China) to construct the treatment plan, including the delineation of gross tumor volume, clinical target volume, planned tumor volume (PTV), and the dangerous organs (including major vessels and spinal cords). An area of 0.5 to 1.0 cm beyond the tumor volume was included in the PTV. The calculated quantity of I-125 seeds that needed to be implanted for D_90_ (the dose to 90% of the volume), when the scope of irradiation was within the scope of 60 to 140 Gy and median dose was approximately 120 Gy, was based on the tumor volume.^[8]^ The I-125 seeds were provided by Beijing HTA Co., Ltd., Beijing, China. The apparent activity of the I-125 sealed seed source was 0.7 mCi, and its half-life was 59.43 days.

### 2.3 The seeds implantation methods

Liquid diet and oral laxatives were given to the patients 1 day before implantation. Fasting and cleaning enema were preoperatively performed at 6 to 8 hours. Oral intestinal indigestible antibiotics were administered before the treatment. The appropriate body posture for the implantation was selected for the intended pathway of puncture. An 18 G (1 G = 0.0678 mm) needle was used to puncture the lesion site in accordance with the treatment plan; the space between needles was 1.0 cm. The space between seeds for an identical needle track was controlled at 1.0 cm. Seeds were implanted 1 by 1 in multiple planes for a distribution with equal spacing. The regulated quantity of seeds used during the implantation was based on the treatment plan and actual situations in the operation. Postoperative validation was performed after seeds implantation is completed. I-125 seeds should be supplemented to achieve the desired therapeutic dose, if the effective dose is not reached or dose loss is present in the tumor. Finally, fasting was advised for 2 to 3 days after implantation, and parenteral nutrition was provided. Drugs to inhibit pancreatic secretion, such as octreotide acetate, were used for 2 to 3 days. Prophylactic use of hemostatic drugs was recommended if small vessels were possibly injured during puncturing. The antibiotics were administrated if the puncture pathway passed through the gastrointestinal tract.

### 2.4 Patient follow-up

Patients were followed up after seed implantation. The side effects and complications were closely monitored. The efficacy evaluation was performed 1 month after implantation. Regular items included physical examination, routine blood test, chest x-ray, abdominal CT scans. Then, a clinical consultation was provided, followed by evaluation every 2 to 3 months or sooner if a new clinical sign or symptom appeared.

### 2.5 Statistical analyses

SPSS version 18.0 statistical software (SPSS Inc., Chicago, IL) was used for all statistical analyses. The actual number (%) was used to represent qualitative data, whereas 
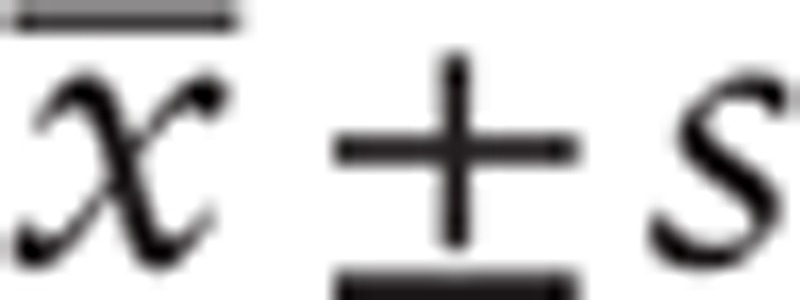
 ± *s* was used to represent quantitative data. Pearson *x*^2^ test or Fisher's exact probability was used to compare the rates between groups for qualitative data. The statistical significance of differences was represented by *P* < .05.

## 3 Results

### 3.1 The baseline characteristics

A total of 78 patients were included in this study, with 53 males and 25 females; the median age was 64 years (range, 44–87 years); the baseline characteristics are summarized in Table 1.

**Table 1:**
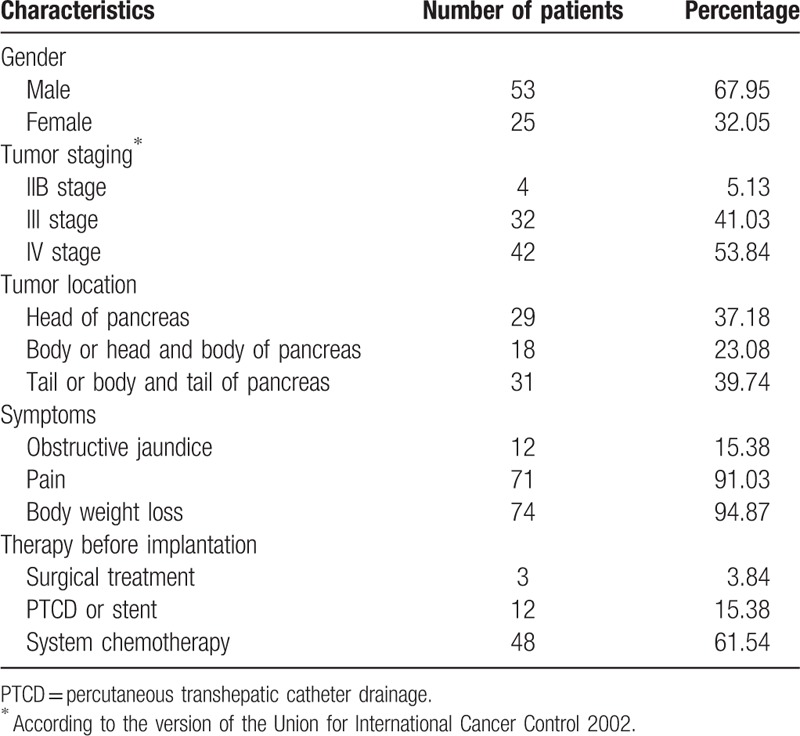
Baseline characteristics of the patients (n = 78).

### 3.2 Side effects and complications following implantation

The side effects after implantation were present in 47 cases (60.26%), including fever in 29 cases (37.18%), abdominal pain in 26 cases (33.33%), and nausea or vomiting in 9 cases (11.54%), among others (Table 2). A total of 18 cases with complications were present (23.08%). The common complications were pancreatitis in 9 cases (11.54%), infection in 4 cases (5.13%), and seeds migration in 2 cases (2.56%). Only 1 patient (1.28%) died of perioperative complication, namely, pyemia as a result of intestinal perforation.

**Table 2:**
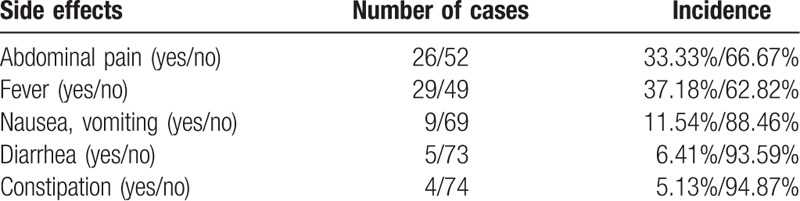
Distribution of side effects following implantation.

**Figure 1:**
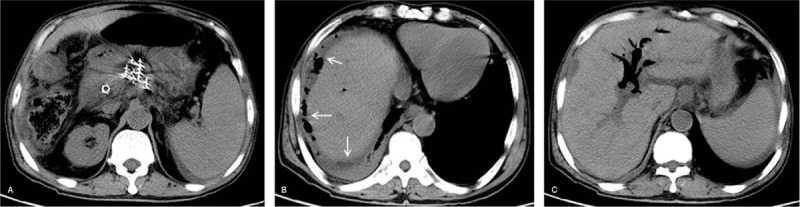
Intestinal perforation, abdominal cavity infection and biliary pneumatosis were complicated in an advanced pancreatic cancer patient, who was treated by iodine-125 seed implantation. (Left) Iodine-125 seeds were implanted under CT-guide. (Center) Abdominal cavity infection resulted from intestinal perforation was revealed (arrow) when CT follow-up was performed on the 7th day after implantation. (Right) Biliary pneumatosis were shown on CT scans.

**Figure 2:**
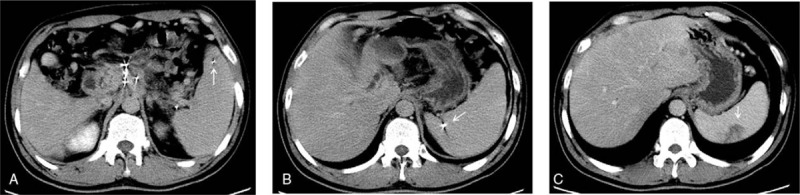
Iodine-125 seeds migrating to spleen and leading to spleen infarction. (Left and Center) The migrated iodine-125 seeds were found in spleen on the CT scans during the follow-up of post-implantation (arrow). (Right) Infarction of spleen resulted from seeds migration was confirmed on the enhanced CT scans (arrow).

According to the treatment plan performed before implantation, the median number of implanted seeds was 27 for all patients. These patients were divided into the Group A and Group B based on this median number. About 40 patients with ≤27 implanted seeds were selected into Group A, whereas 38 patients with >27 implanted seeds were identified as part of Group B. Complications were present in 5 cases (12.50%, 5/40) of Group A, namely, 2 cases of postoperative pancreatitis (5.0%), 2 cases of infection (5.0%), and 1 case of intestinal obstruction (2.50%). Complications were also present in 13 cases (34.21%, 13/38) of Group B, including 7 cases of pancreatitis (18.42%), 3 cases of infection (7.89%), of which 1 case was intestinal perforation combined with abdominal cavity infection and biliary pneumatosis (Fig. 1), 1 case of ketoacidosis (2.63%), and 2 cases of seed migration (5.26%; Fig. 2). The incidence of postoperative complications was lower in patients of Group A than that in patients of Group B (12.50% vs 34.21%, *P* = .032; Table 3).

**Table 3:**
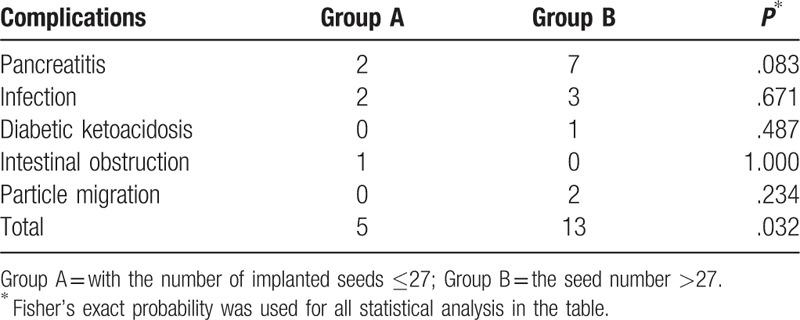
Comparison of incidence of complications between groups with different numbers of implanted seeds.

### 3.3 Number of punctures and complications

A total of 61 patients had the puncture pathway passing through the adjacent organs; complications were present in 12 of 56 patients (21.43%) with the puncture pathway passing through the gastrointestinal tract. No complications were present in the remaining 5 patients with the puncture pathway passing through the liver and spleen, whereas complications were present in 6 of 17 patients (35.29%) with a puncture pathway that did not pass through adjacent organs. No statistically significant differences were found when the incidence of complications was compared between patients with a puncture pathway passing through adjacent organs and those with a puncture pathway that did not pass through adjacent organs (21.43% vs 35.29%, *P* = .201).

The median number of punctures for each patient during implantation was 5 (range, 2–11). Among the 39 patients with ≤5 punctures, complications were present in 8 patients (20.51%). For the 39 patients with >5 punctures, complications were present in 10 patients (25.64%). The difference of the incidence of complications was not significant between the 2 groups (20.51% vs 25.64%, *x*^2^ = 0.289, *P* *=* .591).

## 4 Discussion

The interstitial implantation of radioactive seeds into the site of pancreatic cancer provides the advantage of delivering a high dose of irradiation to the tumor (range 140–160 Gy) during a long term, which drops off sharply outside the local implanted field to achieve the persistent killing of tumor cells, thereby improving the patient's life quality, extending the survival time for patients with advanced pancreatic cancer, and creating significant pain-relieving effect.^[8]^ The efficacy and safety of I-125 seeds implantation in patients with pancreatic cancer have been validated by reports made at individual research centers.^[8]^

The postoperative side effects presented in this study primarily include short-term aggravated pain and gastrointestinal symptoms, such as fever and pernicious vomiting; among these symptoms, postoperative abdominal pain and fever are most common but are seen to improve after symptomatic treatment. More concern should be given to postoperative side effects by alerting to the occurrence of complications when postoperative side effects are present. Reexamination of blood and urinary amylase should be actively performed when short-term postoperative abdominal pain is present. Retest of abdominal CT should be conducted if necessary, whereas active management should be given when pancreatitis is found. With regard to postoperative fever, Wang et al^[9]^ reported that transient fever is present in 36% of the patients after seed implantation, but infection was not observed. The transient fever may be attributed to the “postoperative wound fever”, which frequently occurs in patients underwent major operation due to operative wound and stress reactions, which can be differentiated from infectious fever by routine blood tests and blood cultures. The anti-inflammation should be actively managed to avoid progression toward septicemia and retroperitoneal abscess when signs of infection are present.

The primary complications demonstrated by individual reports are composed of pancreatitis, pancreatic leakage, infection, intestinal obstruction, gastrointestinal perforation, and seed migration, among others; pancreatitis and infection are the most common among these complications.^[10–13]^

In the present study, the overall incidence of pancreatitis after implantation of I-125 seeds is approximately 11.54% (9/78). The incidence of pancreatitis in patients with implantation of minor seeds is not statistically significant compared with those with implantation of major seeds (5.0% vs 18.42%, *P* = 0.083), probably because of the smaller number of cases in the present study. The pathogenesis of pancreatitis caused by the implantation of I-125 seeds can be speculated as follows: Exosmosis of pancreatic fluid is probably a result of the injury of normal pancreatic tissues caused by puncture and seeds radiation. Small amounts of the gastric or intestinal fluid may enter the pancreas if the puncture passes through gastrointestinal tract; consequently, trypsinase is activated. The essential measures for prevention of postoperative pancreatitis include strict preoperative and postoperative fasting, use of somatostatin to inhibit pancreatic secretion, and the avoidance of early or long-term out-of-bed activity. The selection of a proper puncture pathway and accurate puncture of tumor tissues would also be beneficial.

Another major complication in this study is postoperative infection. Puncture into gastrointestinal tract cannot be avoided for most pancreatic puncture, certainly leading to the potential increase of postoperative infection. However, Liu et al^[10]^ reported that the infection rate after implantation of I-125 seeds is significantly lower than that after surgery; no serious outcomes were found in most cases. Most puncture pathways for implantation of I-125 seeds pass through the gastrointestinal tract; thus, the infections are generally caused by enterobacteria. The current results indicate that there is no statistical difference in the incidence of postoperative infection between patients with puncture frequency of ≤5 and those with puncture frequency of >5 (20.51% vs 25.64%, *x*^2^ = 0.289, *P* *=* .591). The puncture frequency is likely not predisposing factor for the occurrence of infection. Apart from the rational selection of the puncture pathway to reduce the puncture frequency, postoperative blood routine retests and prophylactic use of antibiotics should be performed as necessary to prevent postoperative infection.^[8]^

Gastrointestinal perforation is 1 of the critical complications resulting from implantation of I-125 seeds in patients with pancreatic cancer. Therefore, much concern should be given to abdominal symptoms after implantation. Yu et al^[7]^ presented events of intestinal perforation, but fatal events were not found. In the present study, 1 patient with postoperative gastrointestinal perforation accompanied with infection died of pyemia because inadequate concern was given during the early stages of this operation. Preventive measures against gastrointestinal perforation include the selection of a proper puncture pathway, strict fasting, monitoring of electrolytes and the timely relief of abdominal symptoms, such as abdominal distension.

Gastrointestinal obstruction is included as a gastrointestinal-related complication. In the present study, the gastrointestinal obstruction that occurred in our hospital is paralytic ileus, which is associated with multiple injuries of gastrointestinal tract during implantation processes, postoperative use of opioid drugs, long-term lying-in-bed of patients, and electrolytic disorder, among other factors.^[9]^

Migration of I-125 seeds has been mentioned in previous reports, but no serious reactions were induced.^[9,11,12]^ The most common migration pathways for I-125 seeds are the blood vessels and pancreatic duct. In the vascular pathway, the seeds slip into arteries or veins during implantation procedures, mostly into splenic arteries and veins. In the present study, the migration of I-125 seeds into splenic artery and leading to spleen infarction with a size of 2 cm is found in 1 patient. If the seeds are implanted or slipped into pancreatic ducts, they can subsequently enter the intestinal tract and eventually empty out from the bowel. Enhanced CT or magnetic resonance imaging should be performed before implantation to identify the sites of vessels to prevent vascular punctures. Jin et al^[13]^ considered postoperative reexamination of plain abdominal radiographs to be necessary to find the seed migration.

There were some limitations in the present study. Our study was retrospective and the sample size was relatively small. Further studies with expansion of the sample size and prospective randomized controlled trials are needed. Moreover, the lack of control groups existed in this study. Further studies for comparison of different local therapeutic methods in patients with advanced pancreatic cancer with respect to the side effects and complications are warranted.

In summary, the present study indicated that the incidence of postoperative side effects and complications is relatively lower in patients with advanced pancreatic cancer when implantation of I-125 seeds is performed. Comparative analysis revealed that as the volume of pancreatic tumors increased, the quantity of implanted I-125 seeds correspondingly increased in accordance with the treatment plan; the potential risks of occurrence of complications also increased. Implantation of I-125 seeds should be carefully selected for the massive pancreatic lesion. The incidence of complication may be reduced when the puncture frequency is reduced and the puncture pathway is rationally selected. However, the results were not validated by statistical analysis; further analytical study is expected with an increased number of cases. The corresponding incidence of complications may be reduced if preventive measures are taken based on the causes of different complications during the perioperative period.
